# Effects of Hydrogen‐Rich Water on Growth, Redox Homeostasis and Hormonal, Histological and Immune Systems in Rats Exposed to High Cage Density Stress

**DOI:** 10.1002/vms3.70305

**Published:** 2025-03-19

**Authors:** Buket Boğa Kuru, Mustafa Makav, Mushap Kuru, Şükran Yediel Aras, Ebru Karadağ Sarı, Menekşe Bulut, Duried Alwazeer, Fikret Bektaşoğlu, Mükremin Ölmez, Turgut Kırmızıbayrak, Tyler W. LeBaron

**Affiliations:** ^1^ Department of Animal Breeding and Husbandry Faculty of Veterinary Medicine Kafkas University Kars Türkiye; ^2^ Department of Physiology Faculty of Veterinary Medicine Kafkas University Kars Türkiye; ^3^ Department of Obstetrics and Gynecology Faculty of Veterinary Medicine Kafkas University Kars Türkiye; ^4^ Department of Midwifery Faculty of Health Sciences Kafkas University Kars Türkiye; ^5^ Department of Histology‐Embryology Faculty of Veterinary Medicine Kafkas University Kars Türkiye; ^6^ Department of Food Engineering Faculty of Engineering Iğdır University Iğdır Türkiye; ^7^ Department of Nutrition and Dietetics Faculty of Health Sciences Iğdır University Iğdır Türkiye; ^8^ Department of Animal Nutrition and Nutritional Diseases Faculty of Veterinary Medicine Kafkas University Kars Türkiye; ^9^ Department of Kinesiology and Outdoor Recreation Southern Utah University Cedar City Utah USA; ^10^ Molecular Hydrogen Institute Enoch Utah USA

**Keywords:** growth performance | hydrogen‐rich water | molecular hydrogen | oxidative status | stress

## Abstract

**Objectives:**

This study investigated the impact of drinking hydrogen‐rich water (HRW) on growth performance, organ weights, thiol/disulphide homeostasis, oxidative status and some hormonal, histopathological and immunohistochemical changes in rats fed in a restricted housing environment.

**Methods:**

The eight groups (each group [male/female] eight rats) comprised two control, two hydrogen, two stress and two stress + hydrogen. All animals were given feed and water ad libitum for 3 months. Control and HRW group rats were calculated according to weight and housed according to the Guide's housing condition. The stress group and stress + HRW group were housed in half the area of the Guide's housing condition according to their weight. The animal's weekly body weights were measured throughout the study. The animals were sacrificed in accordance with ethical rules. Then, biochemical analyses were performed on thyroid‐stimulating hormone (TSH), free triiodothyronine (FT3), free thyroxine (FT4), cortisol, parathyroid hormone (PTH) and calcium (Ca^2+^), total thiol (TT), native thiol (NT), disulphide, disulphide/TT × 100, disulphide/NT × 100 and NT/TT × 100, malondialdehyde (MDA) and glutathione (GSH). Haematoxylin staining for histopathological and SOD‐2 immunoreactivity was also assessed.

**Results:**

Results showed that live weight gain was higher in the HRW groups than in the stress group. Oxidant status in biochemical analyses decreased in the stress + HRW group compared to the stress group. TSH decreased in the stress group. FT4, cortisol and Ca^2+^ increased in the stress group.

**Conclusions:**

The stress‐related physiological parameters were reduced in the HRW + stress group compared to the stress group. HRW could be suggested when the organism is found in stressful conditions.

## Graphical abstract

Control and HRW groups were housed in 20 × 30 × 45 cages. Stress and stress + HRW groups were housed in 20 × 15 × 45 cages. Weekly weight weighing was done. After 3 months, the animals were terminated, and the results were evaluated by biochemical and histopathological analyses.

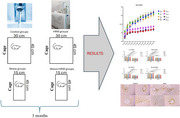

## 1 Introduction

Exposure of organisms to crowded environments can significantly impact body functions, particularly the endocrine system. These effects can include an increase in stress hormones, weakening of the immune system and changes in some metabolic activities (Armario et al. 1984). Crowding can cause an increase in the stress hormones and adrenocorticotropin (ACTH) levels, resulting in lower responses to stimuli and desensitization. This situation can lead to chronic stress and health problems (Yildiz et al. 2007). ACTH triggers the stress response by stimulating the adrenal glands to secrete cortisol. This treatment results in various signs of stress in birds, such as an increase in heart rate and blood pressure, a weakened immune system and impairment in reproductive function (Bray 1993; Bahri et al. 1994; Puvadolpirod and Thaxton 2000). Stress can cause cellular damage, ageing, weakened immunity, stunted growth and development and metabolic disorders. These damages occur due to hormonal imbalance, nervous system inflammation and oxidative stress. Stress leads to the increase in free radical levels in the body (Puthpongsiriporn et al. 2001). The phrase ‘reactive oxygen species’ (ROS) refers to a broad category of oxygen species that exhibit significant reactivity towards biological constituents, causing harm to crucial molecules, such as proteins, lipids and DNA (Conrad et al. 2015).

When ROS levels surpass the cell's ability to detoxify and upset the delicate equilibrium within the cell, oxidative stress takes place. This imbalance causes the accumulation of ROS species, including superoxide radical (O_2_^−^), hydrogen peroxide (H_2_O_2_) and hydroxyl radical (^•^OH) (Halliwell 2006; Winterbourn 2008; Murphy et al. 2011; Hyman and Franz 2012). Cells combat oxidative stress through enzymatic antioxidants, including superoxide dismutase (SOD), catalase and glutathione peroxidase (GPx), as well as non‐enzymatic antioxidants, including thiols, for example, glutathione (GSH). Alterations in enzymatic antioxidants and GSH concentration lead to disruption of the cellular redox homeostasis and raise oxidative stress associated with various diseases (Townsend et al. 2003; Hyman and Franz 2012). Therefore, preventing and managing stress is paramount for overall health and well‐being (Puvadolpirod and Thaxton 2000; Belge et al. 2003).

Molecular hydrogen (H_2_) has a selective antioxidant activity against the most reactive oxidants, that is, hydroxyl radical (^•^OH) and peroxynitrite (ONOO^−^) (Ohsawa et al. 2007). This antioxidant effect is one of more than 37 biological and physicochemical properties reported for H_2_ (Alwazeer 2024). The attributes on the list are as follows: down‐regulator of pro‐inflammatory cytokines, stimulator of energy metabolism, neuroprotector, anti‐inflammatory, antiradical, anti‐cancer, anti‐stress, anti‐apoptosis, anti‐allergic, signalling molecule, redox regulator, gene expression modulator, blood vessel function modulator and myocardial response modulator to ischaemia/reperfusion injury (Alwazeer 2024). The therapeutic advantages of H_2_ were linked to its specific physical, chemical and biological properties (Alwazeer et al. 2021a). Hydrogen administration methods include inhalation, hydrogen‐enriched water, hydrogen‐enriched saline, hydrogen‐enriched eye drops, hydrogen‐enriched bathing, magnesium–water reaction and hydrogen‐generating nanomaterials (Alwazeer 2024). There are many studies revealing the effects of hydrogen‐rich water (HRW) on growth performance (Kuru et al. 2024). HRW is considered a simple method that is applicable indoors and outdoors.

The welfare of laboratory animals and the results of research are seriously threatened by oxidative stress in rats subjected to high cage density stress. Increased oxidative stress markers are strongly associated with a variety of physiological and behavioural stress responses that have been demonstrated to be induced by high cage density. For example, research has shown that rats kept in conditions with high stocking density have higher levels of malondialdehyde (MDA) and nitric oxide (NO), two markers of oxidative stress (Genç 2020). The hypothalamic–pituitary–adrenal (HPA) axis is frequently activated in the physiological mechanisms behind these stress reactions, leading to an increase in the secretion of glucocorticoids like corticosterone. Because elevated corticosterone levels can upset the cellular redox balance and enhance the formation of ROS, they have been linked to oxidative stress (Azman et al. 2018). In addition, many studies have reported that HRW is a particularly powerful antioxidant (Yuan et al. 2018; Alwazeer et al. 2021b; Tian et al. 2021; Kayabaş et al. 2023; Makav et al. 2023; Kuru et al. 2024).

In light of this literature information, the intended hypothesis is to determine the oxidative stress and hormonal activity that develop due to congestion stress. Therefore, the aim of this study is to investigate the potential impacts of HRW on growth performance, organ weights, thiol/disulphide homeostasis, oxidative status, hormonal profiles and histopathological and immunohistochemical parameters of high cage density stress of young rats.

## 2 Materials and Methods

Rats were divided into eight groups, with eight rats in each group. The study used 64 Wistar Albino rats, 32 females and 32 males, weighing 120–220 g and 8 weeks old. Animals were subjected to a 15‐day adaptation period before starting the study. All animals were illuminated during the study for 12 h at night and 12 h during the day. All animals were fed ad libitum according to the feed content in Table S1. The rats were given HRW ad libitum, which was reconstituted every 4 h.

In the study, 20 × 30 × 45 cages were used to house the rats. Rats of control and hydrogen groups were calculated according to weight and housed according to the Guide's housing condition (NRC 2011). The rats of the stress group and stress + HRW group were housed in half the area of the Guide's 18 housing conditions according to their weight.

Groups:

Group I (control [C], female): The rats were fed pellet feed and drinking water for 3 months and received no other treatment.

Group II (control [C], male): Rats were fed pellet feed and drinking water for 3 months and received no other treatment.

Group III (HRW [H], female): The rats were fed pellet feed and HRW for 3 months and received no other treatment.

Group IV (HRW [H], male): Rats were fed pellet feed and HRW for 3 months and received no other treatment.

Group V (stress [S], female): Rats were fed with pellet feed and drinking water for 3 months and subjected to congestion stress.

Group VI (stress [S], male): Rats were fed pellet feed and drinking water for 3 months and subjected to congestion stress.

Group VII (stress + HRW [SH], female): Rats were fed with pellet feed and HRW for 3 months and subjected to congestion stress.

Group VIII (stress + HRW [SH], male): Rats were fed with pellet feed and HRW for 3 months and subjected to congestion stress.

The body weight of rats was measured weekly during the study. Rats were put to death at the end of the trial using the cervical dislocation technique while sedated with ketamine hydrochloride (75 mg/kg) and xylazine (15 mg/kg) intramuscularly. Blood samples were collected for biochemical measurements. The blood samples were centrifuged at 1008 ×*g*, and the serum samples were stored at −20°C until analysis. For histopathological analysis, tissue sampling was performed, and the tissues were stored in a 10% buffered formaldehyde solution. Organ weights were measured by separating the organs from fat and connective tissues after sacrifice with a precision balance.

### 2.1 Preparation of HRW

HRW was prepared using an oxyhydrogen machine (HB‐33 Epoch, Taiwan) by infusing oxyhydrogen into 2 L of drinking water for 30 min at 1.25 L/min. According to Henry's law and the ORP method, the concentration of H_2_ was estimated to be approx. 1 mg/L.

### 2.2 Biochemical Analyses

Thyroid‐stimulating hormone (TSH), free triiodothyronine (FT3), free thyroxine (FT4), cortisol, parathyroid hormone (PTH) and calcium (Ca^2+^) were analysed spectrophotometrically with Abbott Architect c8000 autoanalyser. Total thiol (TT) and native thiol (NT) were measured spectrophotometrically using the TT assay kit and NT assay kit (Rel Assay Diagnostics, Mega Tıp, Türkiye) according to the kit procedure. Disulphide, disulphide/TT × 100, disulphide/NT × 100 and NT/TT × 100 analyses were calculated using TT and NT data (Atalay Mert et al. 2022). GSH analysis was measured using the method of Beutler et al. (1963). MDA analysis was performed according to the method of Yoshioka et al. (1979).

### 2.3 Histological Procedure

Samples of tissue from the kidney, heart and liver were preserved for 24 h in 10% formaldehyde. Following fixation, a routine tissue‐processing protocol was applied. The samples were then embedded in paraffin for histological examination. Haematoxylin and eosin (H&E) staining was performed to assess the general anatomy of the liver, kidney and heart tissues on serial slices that were 5 µm thick.

### 2.4 Immunohistochemistry Procedure

Tissue sections were coated with chromium‐alum gelatine and subjected to the streptavidin–biotin peroxidase method. Phosphate‐buffered saline (PBS, 0.1 M, pH 7.2) was used for all washing steps. Sections were incubated with 3% H_2_O_2_ in 0.1 M PBS for 15 min at room temperature, followed by boiling in citrate buffer solution (microwave oven, 800 W, 10 min). After blocking with Large Volume Ultra V Block solution (10 min), sections were incubated with SOD‐2 (B‐1) primary antibody (Santa Cruz Sc‐133254, 1:500 dilution) for 1 h at room temperature in a humidified chamber. Solutions of streptavidin peroxidase and biotinylated goat anti‐B polyvalent were applied for half an hour at room temperature each. Using the DAB‐H_2_O_2_ substrate, immunoreactivity was seen, and then modified Gill III haematoxylin was used as a counterstain.

To confirm specific immunoreactivity, sections incubated with PBS instead of primary antibody were processed through all steps. Two independent observers assessed the immunostaining intensity and density of target cells using a semi‐quantitative scoring system (no reaction: 0; weak: 1; moderate: 2; strong: 3). All prepared sections were evaluated and photographed using a light microscope (Olympus BX51, Olympus Optical Co. Ltd., Osaka, Japan).

### 2.5 Statistical Analysis

G‐Power 3.1.9.7 was used to do a power analysis before the trial. On the basis of the analysis, the sample size was determined with an effect size (d) of 2.87, test power of 0.95, significant level of 0.05 and findings from the study of Eroğlu, Makav, Fındık Güvendi et al. (2020). Data normality within each group was assessed using the Shapiro–Wilk test. Two‐way ANOVA was conducted for body and organ weight data, as well as biochemical and immunohistochemical parameters, to verify the difference between groups. It was considered significant with a *p* < 0.05. GraphPad 8.1 (San Diego, CA, USA) was used for statistical analysis.

## 3 Results

### 3.1 Body and Organ Weight

**FIGURE 1:**
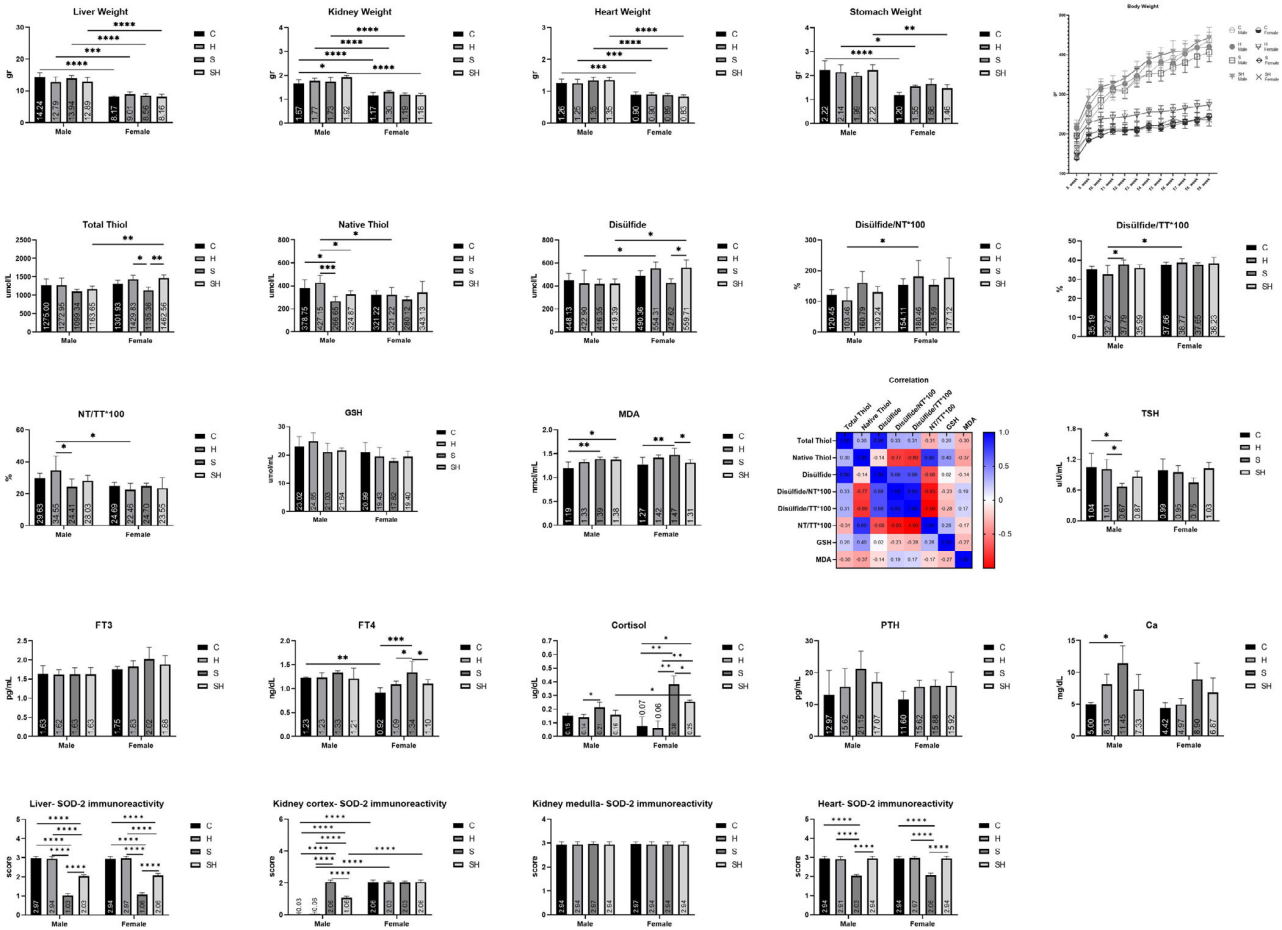
Relative weight gain of rats by weeks. C: control; H: HRW; S: stress; SH: stress + HRW, means and Std. deviation of the eight groups for organ weights. C: control; H: HRW; S: stress; SH: stress + HRW. *p < 0.05, **p < 0.01, ***p < 0.001, ****p < 0.0001. Correlation, means and Std. deviation of the eight groups for total thiol, native thiol, disulphide, disulphide/TT × 100, disulphide/NT × 100, NT/TT × 100, GSH and MDA. *p < 0.05, **p < 0.01, ***p < 0.001. Means and Std. deviation of the eight groups for TSH, FT3, FT4, cortisol, PTH and Ca^2+^. C: control; H: HRW; S: stress; SH: stress + HRW. *p < 0.05, **p < 0.01, ***p < 0.001. Means and Std. deviation of the eight groups for SOD‐2 immunoreactivity. C: control; H: HRW; S: stress; SH: stress + HRW. ****p < 0.0001.

Figure 1 shows the trend in the body weight of rats. Results showed a more significant increase in body weight occurred in male rats than in females for all groups from the second week onwards. In the sex‐related evaluations, the stressed (S) group showed less body weight gain than the other groups for both females and males. The weights of the liver, kidney, heart and stomach significantly increased more in male groups compared to female ones (Figure 1). In addition, the results showed that male kidney weights of stress + HRW (SH) groups were significantly higher than the control group (*p* < 0.05) (Figure 1).

### 3.2 Biochemical Parameters

Biochemically, TT, NT, disulphide, disulphide/TT × 100, disulphide/NT × 100, NT/TT × 100, GSH, MDA, TSH, FT3, FT4, cortisol, PTH and Ca^2+^ levels were analysed. The results of TT, NT, disulphide, disulphide/TT × 100, disulphide/NT × 100, NT/TT × 100, GSH, MDA, TSH, FT3, FT4, cortisol, PTH and Ca are given in Figure 1.

In males, there was no significant difference in TT levels among all treatment groups, including control (*p* > 0.05) (Figure 1). In females, the TT contents were significantly higher in hydrogen (*p* < 0.05) and stress + HRW (*p* < 0.01) groups compared to stress group. When comparing males and females, only females of the stress + HRW group showed significantly higher TT levels than the males of the same group (Figure 1).

Regarding NT levels, there was no significant difference among the different female groups (*p* > 0.05). In male groups, a significant increase in NT was observed for the control (*p* < 0.05) and hydrogen (*p* < 0.001) groups compared to the stress group, whereas the hydrogen group showed higher levels compared with the stress + HRW group (*p* < 0.05). When the males and females were compared, only the male HRW group had significantly higher NT levels than the female (*p* < 0.05) (Figure 1).

Regarding disulphide levels, there was no significant difference between the male groups (*p* > 0.05). In the female groups, the stress + HRW group showed significantly higher disulphide levels than the stress group (*p* < 0.05). When comparing males and females, the female stress + HRW group showed significantly higher disulphide levels than the male stress + HRW group and the male HRW group (*p* < 0.05) (Figure 1).

Regarding the disulphide/TT100 ratio, there was no significant difference between the female groups. In the males, only the stress group had a significantly higher disulphide/TT × 100 ratio than the HRW group (*p* < 0.05). The disulphide/TT × 100 ratio of the HRW group was higher in females than in males (*p* < 0.05) (Figure 1).

Regarding disulphide/NT100 ratios, there was no significant difference between the female and male groups (*p* > 0.05). However, females of the HRW group showed a higher disulphide/NT100 ratio than males (*p* < 0.05) (Figure 1).

Regarding the NT/TT100 ratio, there was no significant difference among the female groups (*p* > 0.05). In the males, only the HRW group showed a higher NT/TT × 100 ratio than the stress group (*p* < 0.05) (Figure 1). However, males of the HRW group showed a higher NT/TT × 100 ratio than females (*p* < 0.05) (Figure 1).

Correlations between TT, NT, disulphide, disulphide/TT × 100, disulphide/NT × 100 and NT/TT × 100 parameters were also evaluated. The findings showed positive correlations between TT and NT, disulphide/TT × 100 and disulphide/NT × 100 (Figure 1). Conversely, a negative correlation was found between TT and NT/TT × 100 and between NT and disulphide.

Regarding GSH, although there was a numerical increase in its levels in the HRW group and stress + HRW group compared to the stress group, this difference was not significant (*p* > 0.05) (Figure 1). Additionally, the GSH levels in males were numerically higher than in females for all groups; however, these differences were not significant (*p* > 0.05).

Regarding MDA, its levels in stress and stress + HRW groups were higher than those in the control group in males (*p* < 0.05). However, in females, its levels were higher in the stress group than in control (*p* < 0.01) and stress + HRW groups (*p* < 0.05) (Figure 1). This finding means that while HRW decreased MDA levels in stressed females, it did not in stressed males. There was no significant difference in MDA levels when females and males were compared.

Concerning TSH, its levels in the control and HRW groups were higher than in the stress group in males (*p* < 0.05), whereas there was no difference in females (*p* > 0.05) (Figure 1). When females and males were compared, no significant difference was found.

Regarding FT3, there was no significant difference in its levels between the treatment groups and between the sex groups (*p* > 0.05) (Figure 1).

Concerning FT4, there was no significant difference in its levels among the males (*p* > 0.05); however, in females, its levels in the stress group were significantly higher than in control (*p* < 0.001), HRW (*p* < 0.05) and stress + HRW (*p* < 0.05) groups. When comparing males and females, the FT4 level was significantly lower in females than in males (*p* < 0.01).

Regarding cortisol, its levels were higher in the stress group than in the HRW group in males (*p* < 0.05) (Figure 1). In the female groups, the stress group had higher cortisol levels compared to the control (*p* < 0.01), HRW (*p* < 0.01) and stress and HRW groups (*p* < 0.05). When comparing males and females, only the female stress + HRW group showed significantly higher cortisol levels than the same group in males (*p* < 0.05).

Concerning PTH, there was no significant difference between the treatment groups and between the sex groups (*p* > 0.05).

Regarding Ca^2+^, its levels were higher in the stress group than the control group in males (*p* < 0.05), whereas there was no significant difference in females (*p* > 0.05). Additionally, there was no significant difference between males and females (*P*>0.05).

### 3.3 Histological Parameters

**FIGURE 2:**
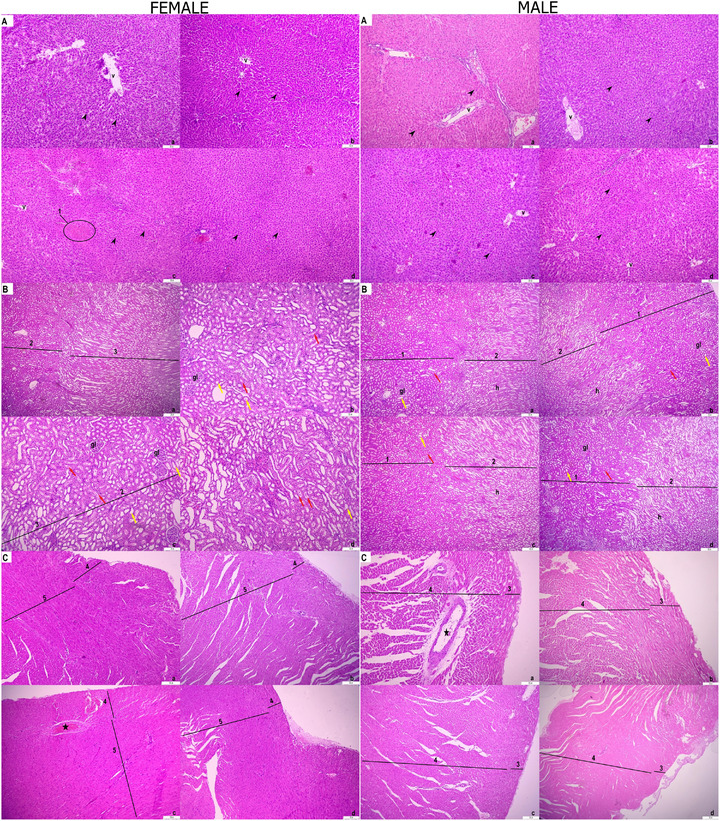
Female rat. (A) Liver tissue; (B) kidney tissue; (C) heart tissue. Control group (a), HRW group (b), stress group (c), stress + HRW group (d). Vena centralis (v), hepatocytes (arrowheads), haemorrhage (1), cortex (2), medulla (3), glomerulus (gl), proximal tubules (red arrow), distal tubules (yellow), loop of Henle (h), epicardium (4), myocardium (5), coronary artery (star). H&E staining. Bar 200 µm. Male rat. (A) Liver tissue; (B) kidney tissue; (C) heart tissue. Control group (a), HRW group (b), stress group (c), stress + HRW group (d). Vena centralis (v), hepatocytes (arrowheads), cortex (1), medulla (2), glomerulus (gl), proximal tubules (red arrow), distal tubules (yellow), loop of Henle (h), epicardium (3), myocardium (4), coronary artery (star). H&E staining. Bar 200 µm.

Histological structures of liver, kidney and heart tissues were determined in all groups of male and female rats (Figure 2). The structure of the vena centralis and hepatocytes was determined in the liver tissue. In addition, haemorrhagic areas were found in the liver tissue in the stress group of female rats (Figure 2A). The cortex and medulla parts of the kidney tissue were determined. Glomeruli, proximal and distal tubules in the cortex region and Henle handles in the medulla region were detected (Figure 2B). In the heart tissue, epicardial and myocardial regions and coronary arteries were determined (Figure 2C).

### 3.4 Immunohistochemical Parameters: SOD‐2 Immunoreactivity

**FIGURE 3:**
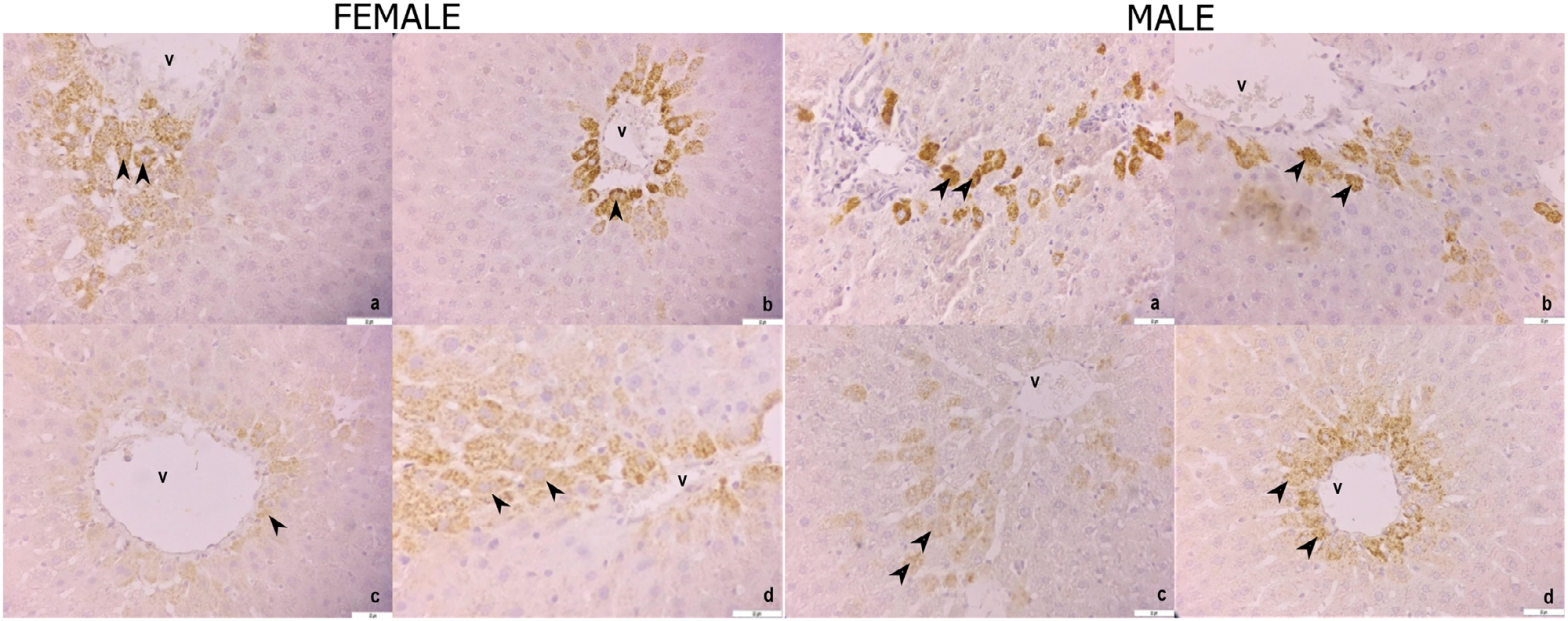
SOD‐2 immunoreactivity in liver tissue of female rats. Control group (a), HRW group (b), stress group (c), stress + HRW group (d). Vena centralis (v), hepatocytes (arrowheads). Bar 50 µm. SOD‐2 immunoreactivity in liver tissue of male rats. Control group (a), HRW group (b), stress group (c), stress + HRW group (d). Vena centralis (v), hepatocytes (arrowheads). Bar 50 µm.

Strong SOD‐2 immunoreactivity was observed in the liver tissues of female rats for the control and HRW groups, weak for the stress group and moderate for the stress + HRW group (Figure 3).

**FIGURE 4:**
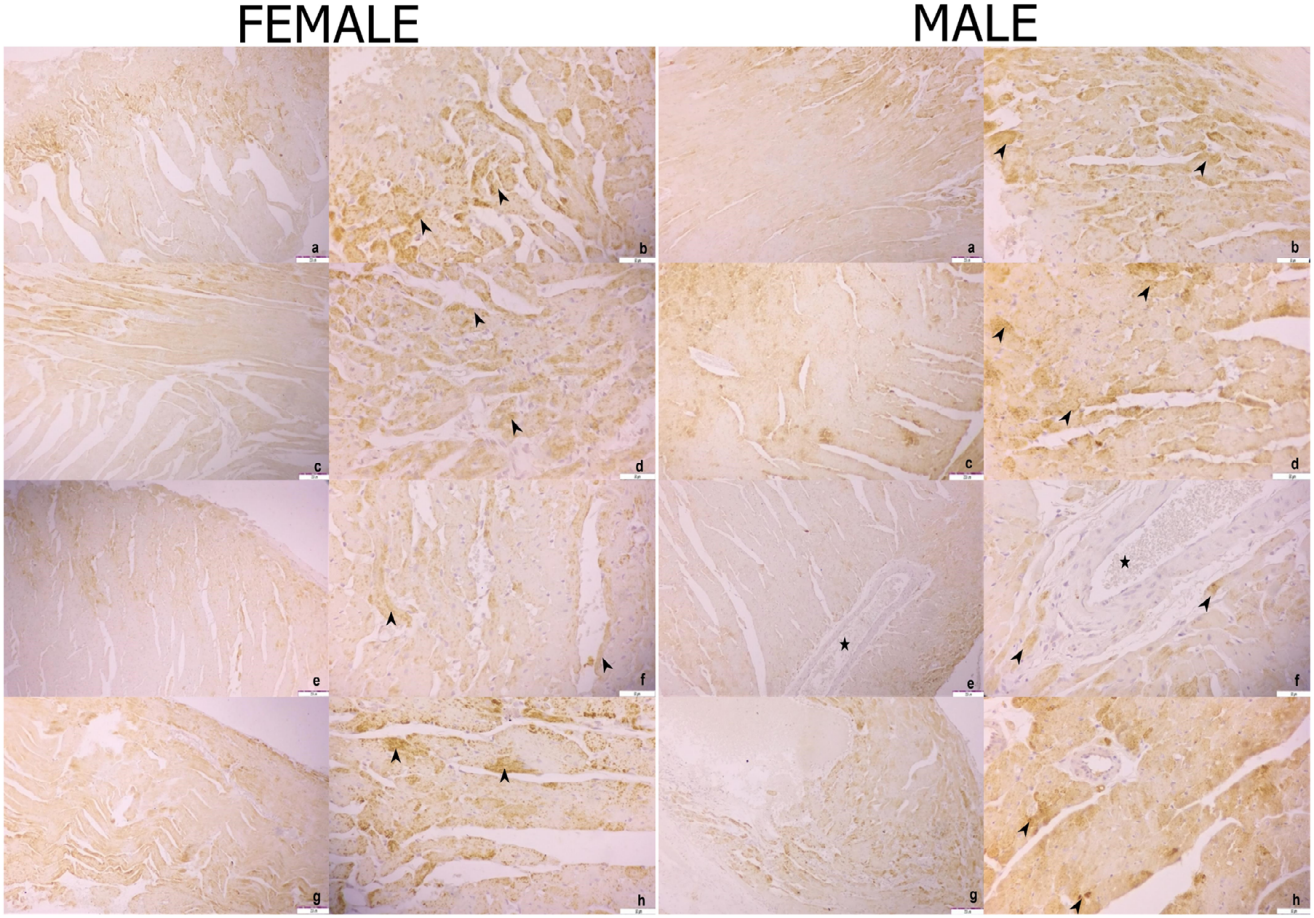
SOD‐2 immunoreactivity in heart tissue of female rats. Control group (a and b), HRW group (c and d), stress group (e and f), stress + HRW group (g and h). Heart muscle cells (arrowheads). Bar 200 µm (a, c, e, g), Bar 50 µm (b, d, f, h). SOD‐2 immunoreactivity in heart tissue of male rats. Control group (a and b), HRW group (c and d), stress group (e and f), stress + HRW group (g and h). Heart muscle cells (arrowheads), coronary artery (star). Bar 200 µm (a, c, e, g); Bar 50 µm (b, d, f, h).

Strong SOD‐2 immunoreactivity was detected in heart tissue for the control, HRW and stress + HRW groups, whereas a moderate SOD‐2 immunoreactivity was observed for the stress group (Figure 4).

Strong SOD‐2 immunoreactivity was observed in the liver tissue of male rats for the control and HRW groups, weak for the stress group and moderate for the stress + HRW group (Figure 3). In the heart tissue, severe SOD‐2 immunoreactivity was detected in the control, HRW and stress + HRW groups, and moderate SOD‐2 immunoreactivity was detected in the stress group (Figure 4).

The evaluation of SOD‐2 scoring between the groups is shown in Figure 1. When SOD‐2 immunoreactivity in the liver was examined, a significant decrease was detected in the stress group compared to the other groups for both males and females. In addition, when the stress + HRW group was compared with the control and stress groups, a significant decrease in SOD‐2 immunoreactivity was noticed. When females and males were compared, there was no significant difference between them. In the heart, the stress group showed significantly less SOD‐2 immunoreactivity than the other groups for both males and females.

## 4 Discussion

The lack of housing space forces individuals to live in cramped environments, which can cause various reactions in the body, even if they are not immediately recognized. One of the most significant reactions is stress, which can lead to various physiological and psychological consequences. According to Rodríguez et al. (2020), for example, people confined in larger residential spaces reported feeling less stressed than people in smaller, more crowded settings. It suggests that having more living space can act as a buffer against the stressors that come with restriction (Rodríguez et al. 2020). Similarly, Halvorsrud et al. (2024) highlight how better home design can provide children with the quiet spaces they need to cope with stressors that come with crowding, such as noise and social interactions. Experimental studies also support the fact that excessive congestion causes stress (Loseva et al. 2015; Pavlova et al. 2024). An animal study has shown that stress can occur due to cage overcrowding, which is similar to the cramped living conditions that some people experience (Yildiz et al. 2007). In another study, it was observed that cortisol levels in the blood increased in the case of stress. This finding revealed the hormonal changes caused by stress in the organism and the possible consequences of these changes (Vélez‐Marín et al. 2012). Cortisol is a steroid hormone secreted by the adrenal glands in response to stress. It leads to various physiological changes and increases the body's resistance to stressful situations (Ok et al. 2024). In the present study, cortisol levels were significantly higher in the cramped group compared to the control group. This finding supports the hypothesis that cage density can cause stress.

Thyroid hormone is well known for regulating growth, metabolism and a host of other bodily processes. T4 and T3 are the two primary hormones that the thyroid gland produces. To preserve appropriate feedback mechanisms and homeostasis, TSH from the anterior pituitary gland, thyroid‐releasing hormone (TRH) from the hypothalamus and T4 function in unison. The common symptoms of hypothyroidism, which is brought on by an underactive thyroid gland, include bradycardia, constipation, exhaustion and weight gain. On the other hand, weight loss, heat intolerance, diarrhoea, fine tremors and muscle weakness are the symptoms of hyperthyroidism, which is brought on by an increase in thyroid gland function. Furthermore, a negative feedback loop involving elevated free T4 and T3 inhibits the release of TSH and TRH (Shahid et al. 2023).

In the present study, the T4 level was significantly increased in the stress group. In addition, there was a decrease in TSH levels. This is thought to be due to cortisol. It has been reported that there is no change in T4‐to‐T3 conversion with increasing cortisol levels, whereas the plasma T3 level decreased (Brown et al. 1991). In the present study, it was noticed that there was a disruption in the conversion of T4 to T3 due to cortisol. Thus, increased T4 levels lead to a decrease in the release of TSH hormone by negative feedback on the pituitary. Therefore, it is thought that a hyperthyroidism‐like condition occurs. This leads to weight loss in particular. Likewise, stress‐induced decreases in both organ weights and body weights. This situation is also directly proportional to bone development. PTH and Ca^2+^ levels of blood increased in the stress groups. PTH is known as the hormone that ensures Ca^2+^ mobilization from bones (Cengiz and Gökçe 2021). Thus, it is noticed that Ca^2+^ in the bones is transferred to the blood, preventing growth in a stressful (cramped) environment. Chronic psychological stress is reported to accelerate biological aging, and oxidative damage is considered the main potential mediator of this process (Aschbacher et al. 2013). However, in the HRW group of rats, both organ and body weights increased, possibly due to the high antioxidant properties of HRW impacting positively on the body's physiology. This may be explained by the fact that molecular hydrogen's unique antioxidant activity (Stone et al. 2022) leads to eliminating excessive oxidative stress and thus decreasing cortisol levels.

It has been demonstrated that oestrogens, especially oestradiol, provide protection against oxidative stress. In postmenopausal women, for example, studies show that oestrogen replacement therapy can lower oxidative stress markers, potentially reducing the risk of cardiovascular illnesses and cognitive decline (Bellanti et al. 2013; Cagnacci et al. 2023). Antioxidant enzymes like catalase and SOD, which are essential for scavenging ROS, are expressed more when oestrogens are present (Strehlow et al. 2003). Additionally, cytochrome P450 enzymes, which have the ability to transform oestrogens into both beneficial and detrimental metabolites, are one of the metabolic pathways that oestrogens have been linked to regulating (Patel and Bhat 2004; Rivera‐Portalatin et al. 2007). On the other hand, in the presence of oxidative stress, androgens, especially testosterone also show neuroprotective qualities. Although the effects may differ on the basis of the cellular setting, studies have shown that testosterone can shield brain cells from oxidative assaults (Tenkorang et al. 2018; Duong et al. 2020). Sex hormones may affect the vulnerability to neurodegenerative disorders by modulating oxidative stress pathways, as evidenced by the varied reactions of male and female neurones to oxidative stress (Tenkorang et al. 2018). When we look at our study, we see that both biochemical data and histopathological data show differences between males and females.

In many studies, it has been reported that the increase in MDA value in various conditions is an indicator of the presence of oxidative stress (Eroğlu, Makav, Adali, et al. 2020; Ölmez et al. 2020; Akyüz et al. 2021; Makav, Dolanbay et al. 2021; 2023; Yıldız et al. 2022). The present study observed a significant increase in MDA value in the congested group. This finding indicates that a cramped environment causes oxidative damage. Thus, oxidative damage should be reduced in the body. It is thought that HRW provided to the treatment groups might reduce oxidative stress, leading to alleviating oxidative damage. Antioxidant defence systems such as thiols (–SH) can also play an important role in the organism. Antioxidants shield tissues and cells from the damaging effects of ROS. Thiols exhibit their antioxidant qualities via a variety of pathways, such as metal ion chelators, radical quenchers and components of the thiol–disulphide redox buffer. Thiol–disulphide exchange reactions convert thiol groups to reversible disulphide bonds quickly and readily under ROS exposure conditions (Çakırca et al. 2022).

Thiol groups of protein and low molecular mass thiols can produce reversible mixed disulphides due to cysteine residue oxidation under oxidative conditions. Thiol groups have the ability to diminish the generated disulphide bonds, hence preserving dynamic thiol–disulphide homeostasis. It is well recognized that this homeostasis is essential for the defence against free radical damage, detoxification, apoptosis, signal transmission, enzyme activity regulation, transcription factors and cellular signalling pathways (Erel and Neselioglu 2014). In the present study, one can assume that the decrease in thiol levels in the stress group can be explained by stress‐related utilization. The high levels of thiols in the HRW‐treated groups, especially in the stress + HRW group, indicate that the molecular hydrogen reduces oxidative stress and decreases the consumption of thiols in stress conditions. This result suggests that molecular hydrogen supports the antioxidant system of the organism. In the present study, it was noticed that the cramped environment causes oxidative damage to rats, which could be reduced by HRW intake.

SOD is the most critical antioxidant defence system against ROS, that is, superoxide anion radicals (O_2_^−^). SOD catalyses the oxidation of one superoxide radical to oxygen molecule (O_2_) and the reduction of another superoxide radical to hydrogen peroxide (H_2_O_2_), a less reactive molecule. H_2_O_2_ formed is then converted to H_2_O and O_2_ by catalase (CAT) and GPx (Aslankoç et al. 2019). Molecular hydrogen has been shown to attenuate oxidative damage by up‐regulating SOD and GPx and increasing their activities and increasing the GSH/GSSG ratio (Barancik et al. 2020). In this study, we observed that stress‐induced SOD activity was immunohistochemically increased in tissues, whereas SOD immunoreactivity was decreased in HRW‐treated groups.

## 5 Conclusions

Oxidative stress increases in rats when housed in a crowded environment, resulting in inadequate growth and development. The HRW used in the present study positively affected both oxidative stress and growth and development parameters. With this study model, drinking HRW can be proposed as a solution to the stress induced by crowded living conditions in humans, drawing an analogy to the stress experienced in animal housing.

## Author Contributions

**Buket Boğa Kuru**, **Mustafa Makav**, **Mushap Kuru**, **Fikret Bektaşoğlu**: experimental applications. **Mustafa Makav**: biochemical analysis. **Buket Boğa Kuru**, **Mustafa Makav** and **Mushap Kuru**, **Mükremin Ölmez**, **Turgut Kırmızıbayrak**, **Tyler W. LeBaron**: data analysis and interpretation, manuscript writing. **Şükran Yediel Aras** and **Ebru Karadağ Sarı**: histological analysis and interpretation. **Duried Alwazeer** and **Menekşe Bulut**: Preparation of hydrogen‐rich water. All authors have read and approved the final article.

## Ethics Statement

Before starting the study, permission was obtained from the Local Ethics Committee for Animal Experiments of Kafkas University (KAÜ‐HADYEK/2021‐152 and KAÜ‐HADYEK/2022‐068).

## Conflicts of Interest

The authors declare no conflicts of interest.

## Supporting information



Supporting Information

## Data Availability

The datasets used during and/or analysed during the current study are available from the corresponding author on reasonable request.
