# Supplementary material for: Effects of Hydrogen‐Rich Water on Growth, Redox Homeostasis and Hormonal, Histological and Immune Systems in Rats Exposed to High Cage Density Stress
Source: Vet Med Sci. 2025 Mar 19;11(2):e70305. doi: 10.1002/vms3.70305 (PMC11920740; doi:10.1002/vms3.70305)
Supplement: Supplementary file 1 — Supporting Information [file VMS3-11-e70305-s001.docx]

**Table S1.** Feed content fed to rats.

| Ingredients | % |
| --- | --- |
| Barley | 28.00 |
| Wheat | 14.00 |
| Wheat Bran | 10.00 |
| Molasses, sugar beet pulp | 4.40 |
| Soybean meal (48% CP) | 13.00 |
| Sunflower meal (26% CP) | 1.00 |
| Marble dust | 1.00 |
| Dicalcium phosphate | 1.00 |
| Calcium carbonate | 1.00 |
| Salt | 0.60 |
| Total | **100** |
| Nutrient analyses |  |
| Crude protein (%) | 20.00 |
| Metabolized energy (Kcal/kg) | 2400 |
| Calcium (%) | 1.16 |
| Phosphorus (%) | 0.74 |
